# Acute Disseminated Encephalomyelitis (ADEM) After Consecutive Exposures to Mycoplasma and COVID Vaccine: A Case Report

**DOI:** 10.7759/cureus.26258

**Published:** 2022-06-23

**Authors:** Heba Mousa, Tanvi H Patel, Idu Meadows, Burcu Ozdemir

**Affiliations:** 1 Internal Medicine, Baptist Health-University of Arkansas for Medical Sciences (UAMS), North Little Rock, USA

**Keywords:** therapeutic plasmapheresis, solumedrol, acute neurological deficit, moderna vaccine, covid-19 vaccine, acute disseminated encephalomyelitis (adem)

## Abstract

Acute disseminated encephalomyelitis (ADEM) is an autoimmune demyelinating disease of the central nervous system, commonly triggered by viral infections or after immunization. ADEM occurrences in adults are rare. Full spectrum of complications is unknown for novel severe acute respiratory syndrome coronavirus 2 (SARS-CoV-2) mRNA vaccines.

A previously healthy 44-year-old female presented to the emergency room (ER) with acute onset of tingling, numbness, and weakness of both lower extremities, urinary retention, blurred vision in right eye, and midline lower back pain. Physical examination revealed bilateral lower extremity weakness 1/5, absent deep tendon reflexes, and decreased sensation. She received the first dose of SARS-CoV-2 vaccine six days prior to presentation to ER. Imaging of her lumbar spine and head were consistent with an active demyelinating plaque consistent with demyelinating disease either multiple sclerosis (MS) or ADEM. The patient was started on SoluMedrol 500 mg IV twice daily for five days. Serological workup and CSF analysis were nonsignificant except for *Mycoplasma pneumonia* IgM, elevated myelin basic protein, and positive IgG, IgA, and IgM. Patient gradually improved and was transferred to rehabilitation. Repeat MRI brain and spine showed improvement in previous lesions. However, she had worsening left eye symptoms that prompted her transfer to another facility for plasmapheresis. Plasma exchange was done for five treatments for ADEM. Patient started noticing improvement in vision and was discharged on steroid taper.

We report a case of a possible association between ADEM and SARS-CoV-2 mRNA vaccine. It should be considered in the differential diagnosis in any case suggestive of acute demyelination after COVID-19 vaccination.

## Introduction

Acute disseminated encephalomyelitis (ADEM) is a neurological manifestation attributed to inflammatory demyelination of the central nervous system. Multiple theories have been proposed to explain the mechanisms of ADEM. One explanation is cross-reaction between viral protein and nervous system (myelin auto-antigens) [1]. Another theory attributes ADEM to inflammation and circulating complexes that follows infection or vaccinations leading to vascular permeability and congestion in the central nervous system [1].

Early onset of manifestations is usually accelerated neurological symptoms depending on the level of brain affected. Symptoms include altered mental status (AMS) with combination of either upper motor neuron (UMN) or lower motor neuron (LMN) lesion or both. Certain cases can be accompanied by generalized symptoms such as fever, body ache, etc. [1]. ADEM occurrences are more prevalent in children than in adults [2]. Before immunization programs, ADEM was commonly attributed to viral or bacterial infection, then later, more ADEM occurrences were reported within eight to 21 days of vaccination [1]. In this case, we reported ADEM post subsequent mycoplasma detection and SARS-CoV-2 mRNA vaccination.

## Case presentation

**Figure 1:**
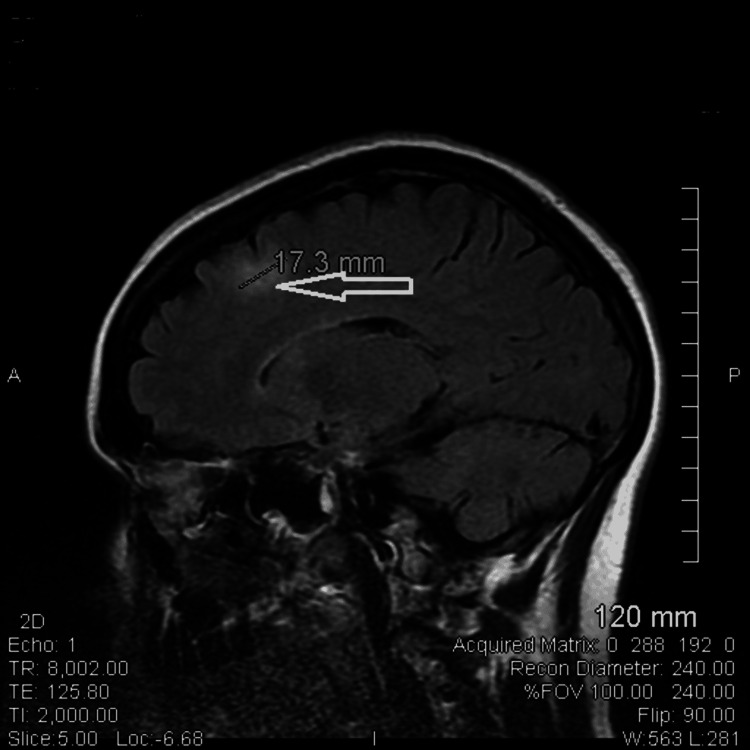
MRI brain with and without contrast shows an enhancing 17.3 mm lesion in the left frontal white matter with surrounding edema.

**Figure 2:**
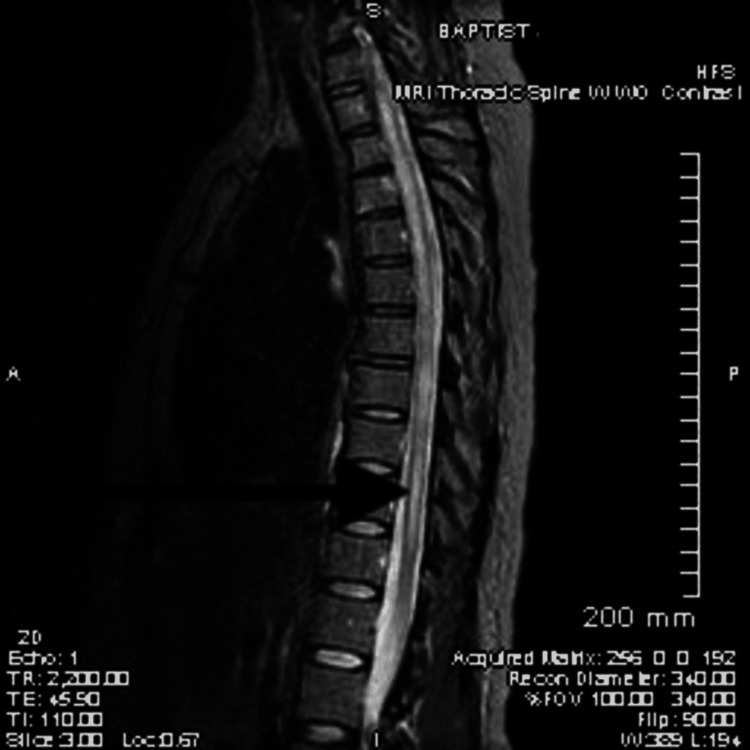
MRI thoracic spine with and without contrast shows diffuse abnormal central cord signal intensity throughout thoracic spine, extending into cervical cord and conus with focal enhancement in left central region at T7-T8 level.

A 44-year-old female with past medical history of anxiety, hyperlipidemia, renal stone, and hypothyroidism came to the emergency room (ER) with complaints of numbness and weakness in both legs. The patient reported that she was completely well, and she takes levothyroxine daily. The previous day, she went to a massage therapist, and the next day she started having tingling and numbness along with weakness in both lower extremities, she also endorsed urinary retention, blurred vision in right eye, and midline lower back pain. Symptoms gradually got worse until she was barely able to move both lower extremities, she got concerned with all these and came to ER. On arrival, her vital signs were stable. Physical examination showed muscle strength 1/5, 0 deep tendon reflexes of bilateral lower extremities, bilateral and equal diminished sensation to touch and pinprick, and sensory level at T3. Gait couldn’t be checked since she wasn’t able to stand, rest of the physical examination was otherwise normal. She denied fever, chills, headache, nuchal rigidity, nausea, vomiting, chest pain, shortness of breath, trouble with speech, facial droop, any trauma, recent infection, or similar complaints in the past. She did receive SARS-CoV-2 mRNA vaccine first dose six days prior to presentation. She reportedly was working in a nursing home, was getting tested for coronavirus disease 2019 (COVID-19) by polymerase chain reaction (PCR)-RNA test twice weekly, and never had a positive result. Initial labs were significant for mild leucocytosis at 12.1 x1,000/mL, serum K of 3.4 mEq/L. T2 MRI lumbar spine with and without contrast was completed, which showed a 12 mm lesion in conus at T11-12 level consistent with an active demyelinating plaque. CT head without contrast showed a 2.4 cm low-density mass in anterior left frontal lobe along with multiple supratentorial and infratentorial lesions consistent with demyelinating disease either multiple sclerosis (MS) or acute disseminated encephalomyelitis (ADEM). Patient was started on SoluMedrol 500 mg IV twice daily for five days, and lumbar puncture and further labs were done (Table 1), and speech and swallow evaluations were done as well; she did not have any signs or symptoms of aspiration. T2 MRI brain, cervical and thoracic spine with and without contrast showed multifocal supratentorial predominantly periventricular lesions with a single lesion in right upper cervical cord and an enhancing 17 mm lesion in left frontal white matter with surrounding edema, again findings consistent with demyelinating disease (Figure 1). Multifocal and diffuse abnormal central cord signal intensity beginning at C3-C4 extending into thoracic spine with focal sparing at C5-C6. Diffuse abnormal central cord signal intensity throughout thoracic spine was favoring ADEM considering recent MRI brain findings (Figure 2). Further workup was done to rule out other potential diagnoses and causes of ADEM.

**Table 1:** Lab results including spinal fluid, serum. VZV: varicella-zoster virus; VDRL: venereal disease research laboratory; RPR: rapid plasma reagin; NMO: neuromyelitis optica; ACE: angiotensin-converting enzyme; CMV: cytomegalovirus; EBV: Epstein-Barr virus; qPCR: quantitative polymerase chain reaction; HSV: herpes simplex virus; COVID-19: coronavirus disease 2019

Test	Result	Reference range
Glucose, CSF	66	40-70 mg/dL
Protein, CSF	98	15-40 mg/dL
RBC, CSF	7	0-5 cells
WBC, CSF	105	0-10 cells
VZV DNA, CSF	Not detected	-
Cryptococcus antigen, CSF	Negative	-
VDRL/RPR, CSF	Non-reactive	-
West Nile Ab IgG, CSF	<1.30	-
West Nile Ab IgM, CSF	<0.90	-
Meningoencephalitis panel, CSF	Negative	-
NMO antibody, CSF	Negative	-
ACE, CSF	6	≤15 p/mol/L/min
Lyme antibody, CSF	Negative	-
Malignant cells, CSF	Negative	-
Myelin basic protein, CSF	10.2	2.0-4.0 mcg/L
IgG, CSF	11.6	0.8-7.7 mg/dL
IgA, CSF	1.8	<0.6 mg/dL
IgM, CSF	1.8	<0.5 mg/dL
Albumin, CSF	59	8.0-42.0 mg/dL
Oligoclonal bands, CSF	No bands	-
CMV IgM Ab	<30	<30 mg/dL
*Mycoplasma pneumonia* Ab, IgG	4.07	≤0.90 mg/dL
*Mycoplasma pneumonia* Ab, IgM	1,943	<770 mg/dL
VDRL/RPR	Non-reactive	-
COVID-19 IgG	0.09	<1.39 mg/dL
EBV DNA qPCR	693	<200 IU/mL
HIV	Non-reactive	-
Flu A	Negative	-
Flu B	Negative	-
CMV qPCR	Negative	-
HSV 1	Negative	-

Oral steroid was started after completing five days of IV steroids, she was started on 60 mg oral prednisone that was tapered by half every three days. Gabapentin and baclofen were started as needed for back pain as well. Patient gradually showed slow improvement, was able to wiggle her toes and later move her feet and was medically stable enough to go to rehab.

She still had bilateral lower extremity weakness, tingling and numbness, blurred vision in right eye, and urinary retention. On day eight since admission, patient had acute worsening blurred vision in left eye (blurry vision of right eye never improved) during her rehab stay and was transferred to another facility for plasmapheresis on day nine from admission.

Plasma exchange was planned for five treatments for acute disseminated encephalomyelitis (ADEM), patient started noticing improvement in visual and urinary symptoms. Repeat T2 MRI brain, cervical and thoracic spine with and without contrast in 14 days showed improvement, previous lesions seemed to decrease in size or resolved. she was discharged on steroid taper with advice to follow up with ophthalmology, urology, and neurology as outpatient. Urinary retention completely resolved, per neuro-ophthalmology, patient likely had optic neuritis which resolved to optic atrophy. Visual field test showed blind spot, ceco-central scotoma in both eyes.

**Figure 3:**
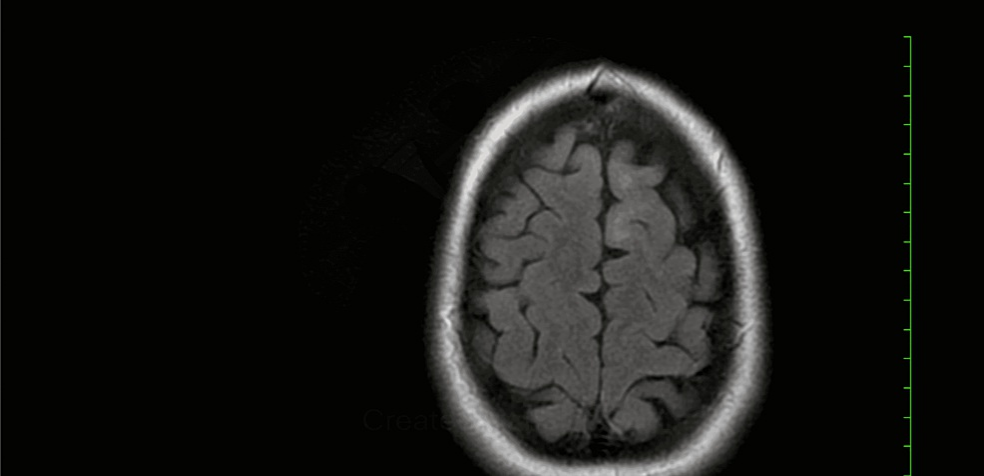
T2 MRI brain with and without contrast in six months shows resolution of demyelinating lesions with no new lesions.

**Figure 4:**
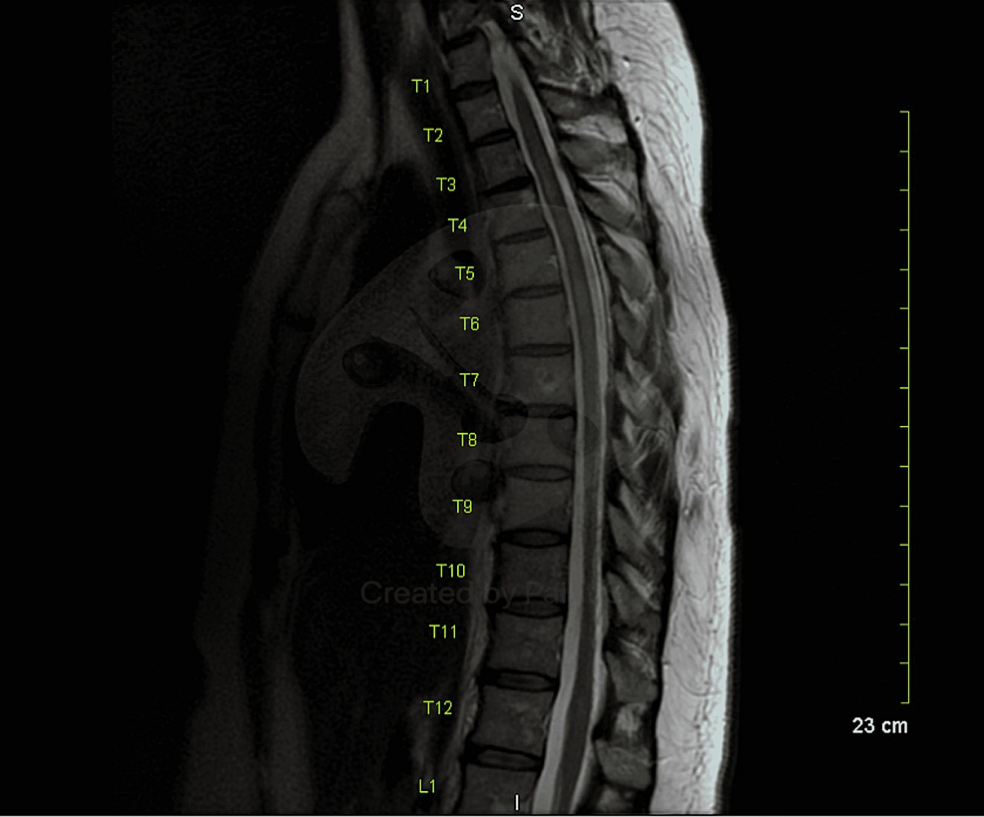
T2 MRI thoracic spine with and without contrast shows resolution of demyelination lesions and no new lesions were found.

Six months later repeat T2 MRI brain and spine with and without contrast were done and showed resolution of previously reported demyelinating lesions and no new demyelinating lesions (Figures 3, 4). Repeat ophthalmological evaluation was stable.

## Discussion

ADEM is an immunologically mediated demyelinating disease triggered by an infection or recent vaccination, eliciting an inflammatory response affecting the central nervous system (CNS). ADEM is more prevalent in children and young adults and is thought to be related to the increased frequency of viral infections and vaccination in that age group [3]. The estimated incidence of ADEM is one in 125,000 to 250,000 individuals every year, it is more common in males than females [1]. International Pediatric Multiple Sclerosis Study Group has proposed the diagnostic criteria for ADEM. The major criteria are clinical attack of CNS demyelinating disease with acute or subacute onset, polysymptomatic neurologic features, and encephalopathy [4]. Impairment of consciousness is present in 46-73% of pediatric patients and in 20-56% of adult cases [1].

After obtaining a detailed history and physical examination, patients with suspected ADEM should get MRI of the brain, cervical and thoracic spine without contrast. Laboratory evaluations of CSF and serum should be obtained mainly to rule out other possible causes, such as infection, multiple sclerosis (MS), neuromyelitis optica spectrum disorder, and myelin oligodendrocyte glycoprotein (MOG) antibody-associated disorder. ADEM presents with typical brain lesions in MRI which are diffuse, ill-defined, symmetric, irregular, and occasionally patchy with areas of homogeneous signal hyperintensities involving both the gray and white matter [5]. Lumbar puncture may reveal abnormalities in 50-80% of patients with ADEM. These findings may include lymphocytic pleocytosis and a slightly elevated CSF protein. More specifically, patients with ADEM are often seen to have an elevated level of myelin basic protein on CSF analysis indicating acute demyelination [6].

Our patient presented with multiple neurological deficits, motor deficit, acute urinary retention, and blurred vision. Although she did not have altered mental status (AMS) which is one of the diagnostic criteria according to the International Pediatric Multiple Sclerosis Study Group, she had demyelinating lesions on MRI brain, cervical and thoracolumbar spine and elevated myelin basic protein on CSF [4].

In case of MS more likely patients present with monosymptomatology and usually optic neuritis is unilateral, they don't have general symptoms or inflammatory markers elevated on labs. CSF analysis can show pleocytosis and positive oligoclonal bands [6]. MRI findings usually have well-demarcated borders, initial imaging can reveal old lesions, and repeat MRI in six months can show new demyelinating lesions. This was not evident in our patient [6].

The patient had a history of recent mRNA COVID vaccine administration and she tested positive for mycoplasma pneumonia IgM and IgG. It would have been beneficial to check for patient's CSF, nasopharyngeal mycoplasma PCR, or CSF mycoplasma culture, to be able to determine if this is confirmed, indeterminate, or possible mycoplasma-related ADEM [7]. Four-fold increase in serum Abs in addition to CSF PCR is confirmatory for acute mycoplasma infection but it is not practical in acutely critical cases [8]. Intrathecal mycoplasma pneumonia Ab detection is an emerging promising test for mycoplasma encephalitis [8]. Positive mycoplasma IgM and IgG is suggestive of an exposure to mycoplasma within the last two months. However, the patient denied any prodromal to suggest recent viral or bacterial infection, and chest examination and imaging were clear. There is literature suggesting possible mycoplasma pneumonia association with ADEM, with no evidence of respiratory infection, evidence of co-infection was detected in 45% and 93% of probable and indeterminate mycoplasma encephalitis, respectively [7].

The timeline suggests consecutive possible infection by mycoplasma and then mRNA COVID vaccine administration. This could represent synergistic effect from the two insults leading to ADEM. Neurological side effects following vaccination are generally mild and temporary, such as fever, chills, headache, fatigue, myalgia, arthralgia, or local injection effects like swelling, redness, or pain. The most catastrophic neurological complication related to SARS-CoV2 vaccine is cerebral venous sinus thrombosis [9]. Another neurological complication is Bell’s palsy which was related mainly to mRNA vaccine administration [10]. Acute transverse myelitis, acute disseminated encephalomyelitis, and acute demyelinating polyneuropathy are other unexpected neurological adverse events that occur as a result of molecular mimicry [11]. Reactivation of herpes zoster following administration of mRNA vaccines has been also recorded [11]. Literature review revealed one reported case with ADEM post-COVID vaccination, later received a SARS-CoV-2 vaccine, including 4 μg inactivated SARS-CoV-2 (Vero Cells; Beijing, China: Beijing Institute of Biological Products Co., Ltd.) two weeks before onset of ADEM symptoms [12].

Treatment options are based on observational studies. First line of treatment for ADEM is high-dose IV corticosteroids, followed by tapered oral prednisolone. Intravenous immunoglobulin (IVIG) is considered in steroid unresponsive patients or in patients who have contraindications to steroids administration [13]. Plasmapheresis (PLEX) is reserved for refractory fulminant cases. As explained above in this case, the patient received IV steroids and five sessions of PLEX when she had worsening visual symptoms.

## Conclusions

Herein, we discussed a case of ADEM following COVID mRNA vaccine administration and mycoplasma detection. ADEM should be considered in the differential diagnosis of any case presented with a suggestive clinical picture after COVID vaccine administration. Moreover, careful immunization history is necessary in any case presented with ADEM. More studies are needed to identify if there is a relation between COVID vaccination and ADEM.
